# Supplementary material for: Bioinformatic Prediction of Signaling Pathways for Apurinic/Apyrimidinic Endodeoxyribonuclease 1 (APEX1) and Its Role in Cholangiocarcinoma Cells
Source: Molecules. 2021 Apr 29;26(9):2587. doi: 10.3390/molecules26092587 (PMC8125001; doi:10.3390/molecules26092587)
Supplement: Supplementary file 1 [file molecules-26-02587-s001.zip › molecules-1166911-supplementary.pdf]

Supplementary Materials

Table S1. The relative expression level of 229 distinct proteins found in the scramble of CCA cell lines

Protein Name	Accession number	[a]Scramble KKU-213A	[b]Scramble KKU-100
2-oxoglutarate dehydrogenase, mitochondrial	A0A0D9SFS3	17.87760	18.38777
5-azacytidine induced 1	B2RN10	17.63603	16.14597
Activating signal cointegrator 1 complex subunit 3	Q8N3C0	14.59243	15.96103
AD 2	Q9Y6Z1	15.15167	14.29617
ADAM metallopeptidase with thrombospondin type 1 motif, 13, isoform CRA_a	A0A024R8G4	19.39700	19.87283
Adenomatous polyposis coli protein isoform t	A0A224AV41	15.61146	16.76630
Adhesion G protein-coupled receptor B2	A2A3C6	17.09320	16.84173
ALS2	A0A2X0SFA0	15.71283	15.95290
Alternative protein ZNF606	L8ECK5	18.01287	17.70510
Amiloride-sensitive sodium channel subunit alpha 1	C5HTY8	15.53853	15.90037
Arf-GAP with Rho-GAP domain, ANK repeat and PH domain-containing protein 3	G5E9Y3	16.08333	16.77900
ARHGEF12	A0A2X0SFF5	16.04597	15.10513
ATPase MORC2	Q9Y6X9	14.79150	17.62440
ATP-binding cassette sub-family A member 10	Q8WWZ4	17.59753	16.79400
ATP-binding cassette sub-family A member 2	A0A087WXK5	13.94003	14.19603
ATP-binding cassette, sub-family C, member 3 isoform MRP3 variant	Q59H05	16.05830	14.61843
Aurora kinase A-interacting protein	Q9NWT8	15.30900	16.01690
BAG family molecular chaperone regulator 3	C9JFK9	15.89267	14.33363
BAH and coiled-coil domain-containing protein 1	F8WBW8	15.41506	17.83900
Beta-klotho	Q86Z14	16.48230	15.33950
Brain my037 protein	Q9H3I9	13.24126	12.97372
Brain specific protein, isoform CRA_a	A0A024R702	18.11290	18.01560
Breast carcinoma amplified sequence 2 variant	Q53HE3	14.73707	15.91413
C10orf54 protein	Q2TA85	14.29267	15.93750
C6orf134 protein	Q8TAV6	16.22963	14.86110
Calcium and integrin-binding protein 1	Q99828	17.53020	15.07003
Calpain-12	Q6ZSI9	18.27563	19.84010
CCR4-NOT transcription complex, subunit 4, isoform CRA_b	A0A024R781	17.79387	17.79763
CDC42	P60953	18.18233	16.82080
Centaurin-alpha 2 protein variant	Q53HQ4	15.02563	15.27843
Centromere protein F	P49454	18.46990	16.87707

Table S1. The relative expression level of 229 distinct proteins found in the scramble of CCA cell lines (cont.)

Protein Name	Accession number	[a]Scramble KKU-213A	[b]Scramble KKU-100
Centrosomal protein 350kDa, isoform CRA_a	A0A024R914	15.37743	16.94347
Centrosomal protein of 120 kDa	Q8N960	18.17633	19.09570
Centrosomal protein of 135 kDa	Q66GS9	16.49937	15.45380
Chromobox homolog 4 (Pc class homolog, Drosophila), isoform CRA_a	A0A0S2Z5B2	13.94140	17.20417
Chronic lymphocytic leukemia up-regulated protein 1	Q5K131	18.04747	16.93763
Cilia- and flagella-associated protein 47	Q6ZTR5	17.62390	16.24830
ciliary rootlet coiled-coil protein 2	H7BZ55	16.08763	16.99510
Cis-aconitate decarboxylase	H7C1Q4	15.31716	15.37867
Coiled-coil domain-containing protein 188	H7C350	14.41050	13.98323
Collagen, type IV, alpha 4, isoform CRA_a	J3KNM7	17.25637	16.62053
Collagen, type VI, alpha 3	D9ZGF2	16.37223	15.43660
COMM domain-containing protein 7	V9GY66	15.81030	14.28113
CREB3 regulatory factor	Q8IUR6	17.62947	17.87150
cyclin-Y-like protein 3	P0C7X3	13.08580	13.41513
Dedicator of cytokinesis 1	B2RUU3	17.06073	17.23113
Dedicator of cytokinesis 8, isoform CRA_a	D3DRH1	18.32153	15.47723
Dedicator of cytokinesis protein 3	Q8IZD9	16.42507	14.05907
Delta globin	A0N071	16.63513	15.81870
Disintegrin and metalloproteinase domain-containing protein 11	K7EKA8	13.82340	15.20453
Dmx-like 2, isoform CRA_c	A0A024R5V2	19.17487	15.25170
DNA excision repair protein ERCC-6	Q03468	14.87870	15.02820
DNA topoisomerase 1	P11387	13.65473	15.18317
DNA-repair protein complementing XP-G cells variant	Q59FZ7	16.87553	16.00773
E1A-binding protein p400	Q96L91	13.67566	16.80097
E3 ubiquitin-protein ligase RBBP6	A0A2R8Y880	17.89767	13.64367
E3 ubiquitin-protein ligase RNF213	A0A0A0MTR7	18.60923	19.21367
E3 ubiquitin-protein ligase TRAF7	Q6Q0C0	14.88397	18.38587
Ectonucleotide pyrophosphatase/phosphodiesterase family member 2	E7EUF1	15.43807	14.42127
EF-hand calcium-binding domain-containing protein 13	Q8IY85	16.58067	14.71837
EMILIN-2	Q9BXX0	15.78950	16.04143
Endothelin-converting enzyme-like 1	O95672	15.84047	18.22840
Envoplakin	Q92817	17.29983	18.32663

Table S1. The relative expression level of 229 distinct proteins found in the scramble of CCA cell lines (cont.)

Protein Name	Accession number	[a]Scramble KKU-213A	[b]Scramble KKU-100
Eukaryotic translation initiation factor 4 gamma, 3 variant	Q59GJ0	24.22767	22.38883
Family with sequence similarity 184, member A	Q6P9G8	15.25993	15.02410
FBLN7 protein	Q8IY13	17.98420	17.12727
F-box/WD repeat-containing protein 11	Q9UKB1	15.32986	13.77667
Fc receptor-like protein 2	Q96LA5	22.58750	22.53563
FEX1	Q0PNF2	15.73710	16.98583
FLJ00077 protein	Q9H7J7	16.64933	13.34683
G protein-coupled receptor 6	E7EP76	15.31173	14.27837
GCN1 general control of amino-acid synthesis 1-like 1 (Yeast), isoform CRA_b	A0A024RBS1	14.81610	12.99043
General transcription factor II-I repeat domain-containing protein 2B	A0A494C1N8	15.99300	15.64023
Glycoprotein receptor gp330/megalin	Q7Z5C1	16.99430	16.54913
Golgin subfamily A member 8S	H3BPF8	16.43907	13.92670
GPI mannosyltransferase 2	Q9NUD9	14.42080	15.28547
GTP-binding protein 1	H0YH10	18.26700	15.61947
Guanine nucleotide-binding protein-like 3	Q9BVP2	15.71447	19.19287
HCFC1	A0A223PQH6	18.57467	16.97147
HCG1990170, isoform CRA_a	D6W4X8	24.53713	20.85010
HCG2009921, isoform CRA_c	Q96MT5	14.43366	16.01797
HCG2036600, isoform CRA_a	D3DNY4	17.74387	18.13080
HCG2039413, isoform CRA_a	A0A024RE15	16.93923	17.15690
HCG401289, isoform CRA_e	A8MT14	13.20563	15.64897
Heat shock 105kDa/110kDa protein 1, isoform CRA_a	A0A024RDQ0	16.22890	15.96347
HERC2-like protein 3	Q9BVR0	14.71810	12.78303
Histone acetyltransferase p300	Q09472	15.70146	17.28517
Histone deacetylase 5	Q9UQL6	15.72247	16.61137
Histone H1.2	P16403	14.06360	14.74320
Histone PARylation factor 1	Q9NWY4	17.84790	16.72517
Histone-lysine N-methyltransferase KMT5C	A0A0D9SF94	14.14720	14.02173
Histone-lysine N-methyltransferase SETD1B	A0A0A0MQV9	16.24203	13.90673
IG c109_heavy_IGHV1-69_IGHD5-24_IGHJ4	A0A5C2GDC2	14.32250	14.08840
IG c1159_heavy_IGHV4-31_IGHD3-22_IGHJ4	A0A5C2GTJ0	13.59333	13.92013
IG c1235_heavy_IGHV3-74_IGHD2-8_IGHJ6	A0A5C2GPC0	13.69417	16.14020

Table S1. The relative expression level of 229 distinct proteins found in the scramble of CCA cell lines (cont.)

Protein Name	Accession number	[a]Scramble KKU-213A	[b]Scramble KKU-100
IG c1531_heavy_IGHV3-74_IGHD3-3_IGHJ4	A0A5C2GVM4	16.59933	18.09440
IG c780_heavy_IGHV3-11_IGHD4-23_IGHJ4	A0A5C2GQG4	16.48320	16.31570
IGH + IGL c482_heavy_IGHV1-69_IGHD4-23_IGHJ3	A0A5C2GBQ8	16.84727	17.51350
IGH c505_heavy__IGHV2-5_IGHD6-19_IGHJ4	A0A5C2GB92	13.49936	15.63530
Inhibitor of Bruton tyrosine kinase	E9PDR5	14.20103	15.63863
Inositol 1,4,5-trisphosphate receptor type 3	Q14573	17.08020	17.53343
Insulin-like growth factor 2 mRNA-binding protein 3	O00425	18.44683	17.82880
Interferon-induced protein with tetratricopeptide repeats 1	P09914	12.31237	12.55710
Interleukin-34	J3QQT3	14.13210	14.31483
Intraflagellar transport 81 homolog (Chlamydomonas), isoform CRA_c	A0A024RBJ1	17.00607	18.40990
Kallikrein-8	A0A0A0MQY9	12.50293	12.89183
Kelch repeat and BTB (POZ) domain containing 4 variant	Q53HN0	15.44260	17.56830
Keratocan	O60938	15.84103	14.07130
KIAA0423, isoform CRA_a	G3XAE9	16.53570	17.97470
KIAA0649, isoform CRA_a	A0A024R8E9	14.87946	16.80693
KIF26A variant protein	B0I1R4	17.37247	17.87057
Kinesin-like protein KIF19	Q2TAC6	16.63303	18.37177
La-related protein 6	Q9BRS8	14.78993	17.18317
Large tegument protein	A0A126LAX2	17.44143	16.35543
Leucine rich repeat containing 24	G3V1D8	17.60197	15.57600
Leucine-, glutamate- and lysine-rich protein 1	J3KP02	13.99303	15.94800
Leucine-rich PPR-motif containing	E5KNY5	18.22730	15.60623
Lymphocyte antigen 75	O60449	17.46047	16.89870
Lysine-specific demethylase 6B	O15054	15.78803	14.22957
Matrix metallopeptidase 15 (Membrane-inserted), isoform CRA_a	A0A024R6U8	15.80653	14.85597
Matrix-remodelling associated 8, isoform CRA_a	A0A024R0A0	13.47153	16.93940
Metastasis-suppressor KiSS-1	A0A0D9SES6	17.80073	16.97213
Microtubule-associated tumor suppressor candidate 2	Q5JR59	18.45887	16.33460
Molybdenum cofactor synthesis 1, isoform CRA_c	A0A024RD17	14.27760	14.20430
MORC family CW-type zinc finger protein 4	Q8TE76	15.35043	16.66670
Mucin 5AC, oligomeric mucus/gel-forming	A7Y9J9	15.19480	17.36117
Mucin-16	Q8WXI7	16.23690	16.68887

Table S1. The relative expression level of 229 distinct proteins found in the scramble of CCA cell lines (cont.)

Protein Name	Accession number	[a]Scramble KKU-213A	[b]Scramble KKU-100
Myosin-10	P35580	16.25173	16.91530
Myotubularin-related protein 6	Q9Y217	15.29543	14.81380
N-acetylaspartate synthetase	Q8N9F0	13.55867	13.87167
NAD-dependent protein deacetylase sirtuin-3, mitochondrial	E9PM52	14.40500	14.95453
NADH dehydrogenase (Ubiquinone) 1 beta subcomplex, 10, 22kDa, isoform CRA_b	A8K761	13.96456	12.50590
Neurobeachin	Q5T321	15.61820	14.75657
Neurofilament heavy polypeptide	P12036	19.27670	17.23753
Neurogenic locus notch homolog protein 4	A0A1U9X943	15.20473	15.55113
Neuroligin 3 isoform A	X5DNV3	16.57070	18.54673
NMDA receptor regulated 1-like	A4FU51	14.40600	16.02517
NOELIN3_V2	Q6IMJ0	16.00263	15.12617
NRAP protein	A0AVL2	17.68630	17.09530
Nuclear factor 1	Q7Z3K7	15.87430	15.30123
Nuclear protein MDM1	Q8TC05	15.67590	15.73000
Nuclear receptor-interacting protein 3	E9PIW3	18.00467	17.19533
Nucleolar protein 8, isoform CRA_a	A0A024R250	14.18330	17.07353
Olfactory receptor 1A1	Q9P1Q5	18.71137	20.03993
PDZ domain-containing RING finger protein 4	Q6ZMN7	16.82587	14.73747
Pecanex-like protein 2	A6NKB5	16.40663	16.47937
Phosphatidylinositol 4-kinase alpha	P42356	14.30720	15.06517
PLA2R1 protein	B7ZML4	16.47977	14.66623
Pleckstrin homology domain-containing family G member 1	A0A2R8Y2B1	14.63725	14.47950
PLEKHG3	A0A2X0SFH4	18.76337	18.87670
Postreplication repair protein hRAD18p variant	Q53H10	18.58957	19.58557
Pre-mRNA-processing factor 40 homolog A	O75400	17.30100	16.85277
PREX1	A0A2X0SFH1	16.83563	16.29473
Probable ATP-dependent RNA helicase DDX6	P26196	13.92507	15.68617
Programmed cell death 11, isoform CRA_a	A0A3B3IUD7	16.57407	18.52967
Prohibitin	D6RBK0	13.96413	14.79397
Protein Daple	Q9P219	15.43177	17.67827
Protein dopey-1	Q5TA12	14.01883	16.26517
Protein FAM131C	Q96AQ9	12.22923	13.09577

Table S1. The relative expression level of 229 distinct proteins found in the scramble of CCA cell lines (cont.)

Protein Name	Accession number	[a]Scramble KKU-213A	[b]Scramble KKU-100
Protein mono-ADP-ribosyltransferase PARP4	Q9UKK3	16.54940	15.60237
PURA protein	Q2NLC9	17.27140	17.25970
RAD50-interacting protein 1	Q6NUQ1	14.95506	12.60600
Ras association (RalGDS/AF-6) and pleckstrin homology domains 1, isoform CRA_b	C9K0J5	15.56210	14.15890
Ras protein-specific guanine nucleotide-releasing factor 1, isoform CRA_b	Q8IUU5	15.53436	17.56273
Retinal-specific phospholipid-transporting ATPase ABCA4	P78363	15.82810	16.77223
Retinitis pigmentosa 1-like 1 protein	Q8IWN7	16.67670	14.04800
Rho guanine nucleotide exchange factor 12	E9PMR6	16.54123	15.66653
Rho guanine nucleotide exchange factor 17	H0YFZ8	20.57300	17.17850
Rhodopsin	V9H017	17.62013	18.56350
Ribosomal protein L15	E7ENU7	16.84987	14.67147
RNA-binding protein 25	P49756	16.56587	17.44307
Rootletin	Q5TZA2	18.92520	16.37253
RRP12-like protein	Q5JTH9	16.64320	14.70807
SAPS domain family, member 3, isoform CRA_c	A0A024R5F3	15.86840	16.32363
SARDH protein	Q05BT1	15.34596	14.85590
Serine/threonine-protein kinase MRCK gamma	Q6DT37	16.92260	16.37440
Serine/threonine-protein kinase ULK4	Q96C45	16.22613	14.61367
SLC12A2 protein	B7ZM24	15.55646	15.77110
SOGA	F2FB34	16.84667	17.00327
Solute carrier 16 (Monocarboxylic acid transporters), member 8, isoform CRA_a	A0A024R1N8	17.47080	17.54893
SOS1	Q53SF8	14.81297	14.99180
Splicing factor, proline- and glutamine-rich	Q2I379	13.24550	14.87215
SPRY domain containing 3, isoform CRA_b	A0A024RAX4	19.42803	18.80773
Sterile alpha motif domain-containing protein 9-like	Q8IVG5	13.75997	15.12553
SUMO-specific isopeptidase USPL1	Q5W0Q7	15.11830	14.59143
SWI/SNF complex subunit SMARCC2	F8VXC8	14.42036	15.65633
Taste receptor type 2 member 46	P59540	12.54436	16.86867
T-cell lymphoma invasion and metastasis 2 variant	Q59EY7	16.10230	16.28703
Testis tissue sperm-binding protein Li 71n	A0A140VKF4	15.39276	13.73707
Threonine--tRNA ligase 2, cytoplasmic	A2RTX5	17.90647	17.04920
Thrombospondin type-1 domain-containing protein 7A	A0A087WY08	17.01590	15.40557

Table S1. The relative expression level of 229 distinct proteins found in the scramble of CCA cell lines (cont.)

Protein Name	Accession number	[a]Scramble KKU-213A	[b]Scramble KKU-100
Thyroxine-binding globulin	P05543	16.44707	18.77900
TNRC4 protein	Q9BTF3	16.33397	17.22477
Trans-3-hydroxy-L-proline dehydratase	Q96EM0	14.12070	13.77217
Transmembrane protein 229B	G3V2T8	14.82793	12.89740
Trichohyalin	Q07283	16.68580	16.72860
Trinucleotide repeat containing 15, isoform CRA_a	A0A024R4A5	13.84173	14.66457
TRPC4 protein	Q3KR50	17.21833	16.11907
Truncated platelet membrane glycoprotein Ib beta	A0A140GX62	13.60433	14.77530
Truncated RHCE	J9ZZ28	18.99867	18.10427
TUBGCP6 protein	B2RWN4	16.26080	16.56540
Tubulin tyrosine ligase-like family, member 1, isoform CRA_a	A0A024R4U6	13.11090	15.70617
Tumor rejection antigen (Gp96) 1 variant	Q59FC6	17.29333	18.52753
Tyrosine-protein kinase BAZ1B	Q9UIG0	15.19106	17.72040
U3 small nucleolar RNA-interacting protein 2	O43818	16.73207	14.76257
Uncharacterized protein CAD	Q53SY7	17.11370	13.54680
Uncharacterized protein DKFZp686A1195	Q6MZU1	15.03130	16.42427
Uncharacterized protein DKFZp686L0869	Q7Z3U5	14.45300	17.45910
Uncharacterized protein DKFZp686L2367	Q63HJ5	17.28743	17.08380
Uncharacterized protein EPAS1	Q53SM6	15.06363	14.10163
Uncharacterized protein KIAA1841	Q6NSI8	14.40963	15.84723
Unconventional myosin-Va	A0A087WY00	17.21363	15.56977
Vacuolar protein sorting-associated protein 13B	Q7Z7G8	15.46263	16.61873
Vacuolar protein sorting-associated protein 13C	Q709C8	16.45590	18.08480
Vitamin D binding protein protein	Q6LDC6	22.42233	20.99427
von Willebrand factor A domain-containing protein 5B2	E9PF42	15.77030	16.46133
YY1AP-related protein1	A4PB67	15.91973	16.14080
Zinc finger and BTB domain-containing protein 46	Q86UZ6	15.81593	14.15240
Zinc finger and SCAN domain-containing protein 20	P17040	18.36460	16.58563
Zinc finger MYM-type protein 6	O95789	13.46780	16.11133
Zinc finger protein 268	F5H467	15.31090	16.09290
Zinc finger protein 40	F5H212	15.72953	16.81953
Zinc finger protein 42 (Myeloid-specific retinoic acid-responsive), isoform CRA_a	A0A024R4R6	17.47743	15.90940

Table S1. The relative expression level of 229 distinct proteins found in the scramble of CCA cell lines (cont.)

Protein Name	Accession number	[a]Scramble KKU-213A	[b]Scramble KKU-100
Zinc finger protein 536	K7EQN6	14.72180	16.48410
Zinc finger protein 579	Q8NAF0	15.43530	17.13520
zinc finger protein 726P1	Q15940	14.38427	15.56697
Zinc finger protein 804A	B9EGN2	15.25293	16.16153
Zinc finger RNA-binding protein	Q96KR1	15.07440	16.33907
ZMAT1 protein	A7MD47	16.62973	16.62247

[a]Values represent the log2 fold change of precursor signal intensities in particular scramble KKU-213A compared to siRNA KKU-213A detected by LC-MS/MS

[b]Values represent the log2 fold change of precursor signal intensities in particular scramble KKU-100 compared to siRNA KKU-100 detected by LC-MS/MS

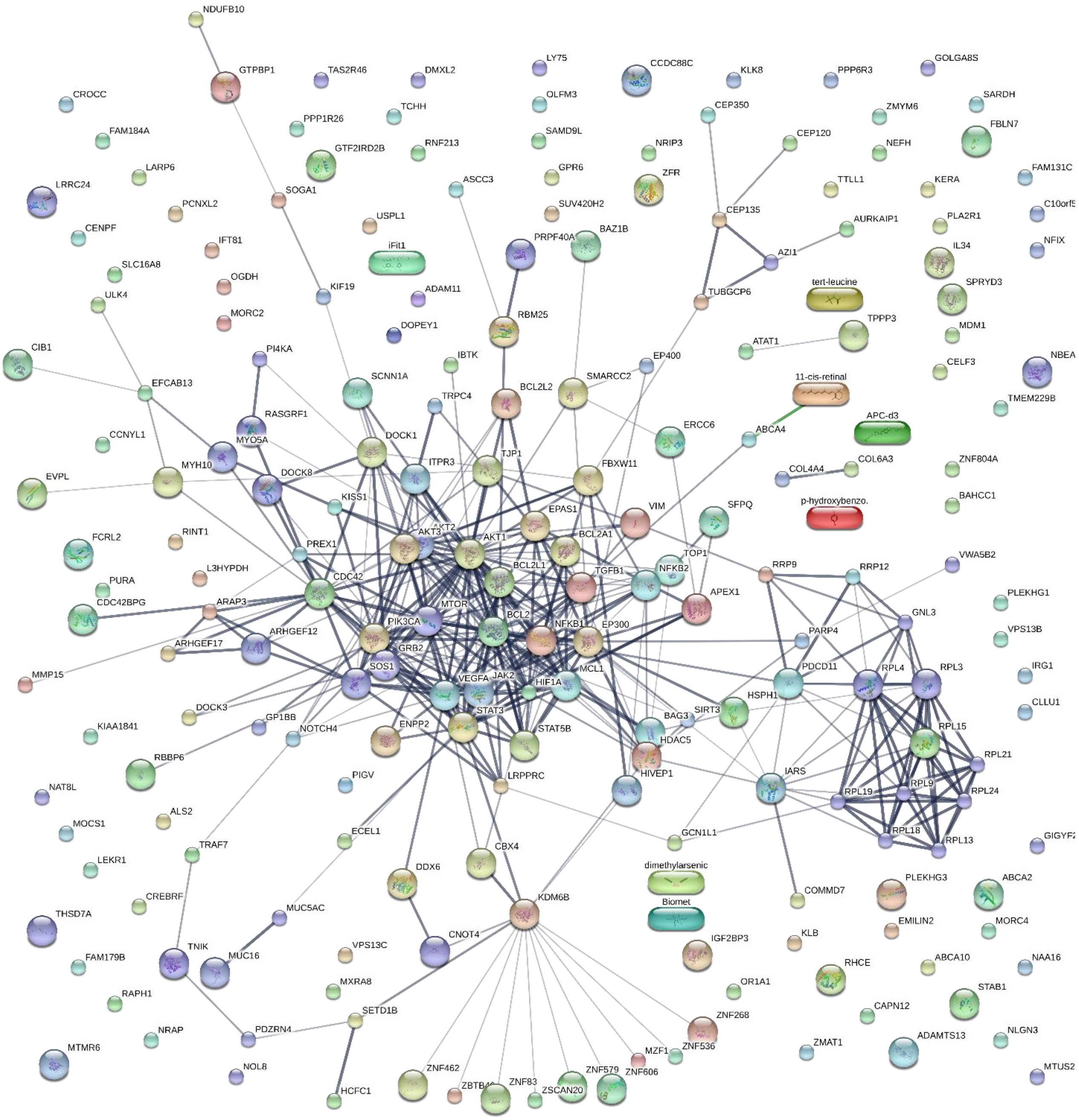

Figure S1. Protein interaction networks of APEX1 and the related proteins of scramble CCA cells.
